# Perspective on Schwann Cells Derived from Induced Pluripotent Stem Cells in Peripheral Nerve Tissue Engineering

**DOI:** 10.3390/cells9112497

**Published:** 2020-11-17

**Authors:** Zhong Huang, Rebecca Powell, James B. Phillips, Kirsten Haastert-Talini

**Affiliations:** 1 Institute of Neuroanatomy and Cell Biology, Hannover Medical School, 30623 Hannover, Germany; huang.zhong@mh-hannover.de; 2 Center for Systems Neuroscience (ZSN) Hannover, 30559 Hannover, Germany; 3 Department of Pharmacology, UCL School of Pharmacy, 29-39 Brunswick Square, London WC1N 1AX, UK; rebecca.powell.17@ucl.ac.uk; 4 UCL Centre for Nerve Engineering, University College London, London WC1E 6BT, UK

**Keywords:** Schwann cells, induced pluripotent stem cells, peripheral nerve, regenerative medicine

## Abstract

Schwann cells play a crucial role in successful peripheral nerve repair and regeneration by supporting both axonal growth and myelination. Schwann cells are therefore a feasible option for cell therapy treatment of peripheral nerve injury. However, sourcing human Schwann cells at quantities required for development beyond research is challenging. Due to their availability, rapid in vitro expansion, survival, and integration within the host tissue, stem cells have attracted considerable attention as candidate cell therapies. Among them, induced pluripotent stem cells (iPSCs) with the associated prospects for personalized treatment are a promising therapy to take the leap from bench to bedside. In this critical review, we firstly focus on the current knowledge of the Schwann cell phenotype in regard to peripheral nerve injury, including crosstalk with the immune system during peripheral nerve regeneration. Then, we review iPSC to Schwann cell derivation protocols and the results from recent in vitro and in vivo studies. We finally conclude with some prospects for the use of iPSCs in clinical settings.

## 1. Introduction

Schwann cells (SCs) develop from the neural crest [1] and are important for peripheral nerve development, function, and repair after injury. This makes SCs or SC-like cells a valuable option for tissue engineering approaches directed towards replacement of injured peripheral nerve tissue. To our knowledge, this review is the first to focus on differentiation of SCs from induced pluripotent stem cells (iPSCs), and what this implies for the field of peripheral nerve regenerative medicine. During development, they become associated with developing axons through a process of radial sorting [2,3]; SCs wrap around larger developing axons and produce myelin as they mature [2]. This myelin sheath around the axons insulates and increases the speed of conduction of electrical impulses between the central nervous system (CNS) and sensory and motor target organs. Schwann cells associated with smaller-diameter axons are non-myelinating or Remak SCs and are likely to have a metabolic and mechanical support function in mature nerves [2].

Schwann cells are not only essential for the normal functioning of peripheral nerves but also for their regeneration after injury [4]. Peripheral nerve injury induces a sequence of events in SCs that is essential for the regeneration process. After injury, Wallerian degeneration in the distal nerve end clears debris [5,6]. Upon losing contact with the collapsed axon, SCs begin to upregulate the transcription factor c-Jun which initiates their transformation to repair SCs [7]. These cells undergo myelinophagy (autophagy of myelin and myelin debris), become proliferative, and elongate to form tracts called bands of Büngner [5,6,8,9]. Myelinophagy by repair SCs, together with debris removal by invading macrophages, is crucial for a timely initiation of further repair processes (see next paragraph). What is also upregulated in repair SCs is the expression of proteins contributing to a pro-regenerative environment for regenerative axon growth [8], e.g., nerve growth factor (NGF) and glial cell-derived neurotrophic factor (GDNF).

## 2. Schwann Cells in the Injured Peripheral Nerve

Within the peripheral nerve injury environment are a multitude of intercellular interactions, many of which center around the repair SCs and are essential for axonal regeneration. Repair SCs not only interact closely with the regenerating axon but also with macrophages, neutrophils, endothelial cells, and fibroblasts in and around the nerve bridge [10]. Interaction takes place through direct contact or indirectly through neurotrophic factor and cytokine release. Macrophage and repair Schwann cell interactions have been studied extensively. Resident macrophages respond to hypoxia in the nerve bridge by upregulating VEGF-A (vascular endothelial growth factor A) release which triggers the formation of new blood vessels across the nerve bridge [11]. These blood vessels provide oxygen to the damaged area and act as tracks for the repair SCs infiltrating from both ends of the nerve gap. Expression of the Sox2 transcription factor in repair SCs induces Robo1 receptor expression at the cell surface which binds to the Slit3 ligand released from macrophages surrounding the nerve bridge. This interaction is repellent and forces the repair SCs to remain on trajectory in the nerve bridge, supporting and directing the regenerating axons [12,13]. Axon pathfinding is defective in Slit3+/−, Slit3−/−, and Robo1+/− mice, as well as when Sox2 is knocked out in SCs [12]. Sox2 expression also results in an increase in the number of infiltrating macrophages in the area [14], alongside the release of the chemokine CCL2 which acts on the macrophage receptor CCR2 [15]. Macrophages phagocytose debris in the injury area, and help induce neutrophils to do the same via the release of the chemokine CXCL1/2 which binds to CXCR2 on neutrophils [16]. The remyelination of axons and transformation of repair SCs (back) to myelinating SCs later in the regeneration process is also mediated by macrophages. When macrophage numbers were reduced one week post-injury in vivo, there was a significant reduction in remyelinated axons despite an increase in the number of repair SCs and immature Sox2+ SCs [17]. The macrophage-secreted ligand Gas6 is essential for this transformation of repair SCs and axon remyelination [17].

Despite macrophages initiating the formation of guiding blood vessels across the nerve bridge, repair SCs will not migrate along these without altering their behavior. This behavioral switch is initiated by the release of ephrin-B from fibroblasts. Ephrin-B activates EphB2 receptors on SCs, upregulating Sox2 expression in the repair SCs [18]. Another role of Sox2 is seen here whereby expression induces the relocalization of N-cadherin on the SCs surface to cell–cell junctions, allowing the repair SCs to migrate across the nerve bridge in cords of cells [18]. When EphB2 is knocked out, axon regeneration is disorganized as seen too in Sox2 knockout mice [12,18]. Fibroblasts are also affected by the transformed repair SCs, which trigger the release of CSF1 cytokine and in turn activate macrophages and additionally target them towards the injury area [19,20].

Repair SCs and regenerating axons directly interact via N-cadherin/NCAM [21], with repair SCs moving across the nerve bridge ahead of the regenerating axon and so acting as a strong guidance cue [22] while they interact with endothelial cells through intercellular contact. Repair SCs also respond to chemokines and cytokines such as TGFβ1 which binds to TGFβ type 1 receptor on the repair SCs surface to induce expression of the collagenase enzymes (or matrix metalloproteinases) MMP2 and MMP9 [23]. These allow repair SCs to become invasive and adopt a mesenchymal-like phenotype to bridge the nerve gap [24].

When the response of SCs is impaired, there is a clear reduction in axon regeneration, indicating the importance of having these cells in cell therapy-based treatment of peripheral nerve injury. Aging SCs in mice fail to upregulate c-Jun after injury, leading to a reduced ability of myelinating SCs to transform into repair SCs, less effective myelin clearance, and reduced recruitment of macrophages that ultimately results in impaired axon re-growth after injury [25]. Aging mouse SCs demonstrate reduced expression of nerve growth factor receptor (NGFR) as well as growth factors such as brain-derived neurotrophic growth factor (BDNF) [26], which is, among other growth factors, important for functional recovery after peripheral nerve injury [27]. Impaired macrophage recruitment is also seen in aged mice due to reduced expression of chemokines such as CCL2 released from repair SCs, which impacts myelin clearance alongside the reduced phagocytic abilities of repair SCs and macrophages [28]. Chronic inflammation in the nerves of aged mice (“inflammaging”) results in reduced axonal regeneration after injury [29]. Similarly, when long acellular nerve allografts are used to repair an injury in rats, the survival of host SCs is impacted, causing them to become senescent over time and preventing successful axon regeneration [30]. The interactions between repair SCs and axons and macrophages demonstrated to be severely impacted in these studies, resulting in limited regeneration.

From the paragraphs above, it is obvious that SCs play a crucial role in orchestrating the peripheral nerve regeneration process. This in turn keeps them at the forefront of candidates for regenerative cellular replacement approaches.

## 3. Purification and Culture of Primary Human Schwann Cells

During research and development stages of cell therapies for future clinical application, relatively small numbers of cells can be sufficient for generating reproducible results, costing relatively little in funding and resources. Progressing to in vivo studies and clinical trial phases requires a huge expansion in the number of cells, as well as the introduction of additional processes such as automation and cryopreservation and progression to costly good manufacturing practice (GMP) laboratory environments [31,32]. Human cells from the nervous system (central and peripheral) are ethically and technically particularly difficult to obtain as well as to expand and maintain in vitro. This also applies to primary human SCs, the focus of the current review, which are isolated from peripheral nerves, presenting the first issue in obtaining a sufficient source of cells due to the limited donor tissue availability and invasiveness of the procedure. Different issues exist in the isolation of SCs directly from PNS tissues and establishing high-purity SCs in culture [33]. Primary human SCs purification and culturing protocols have been refined to increase SCs yield and reduce contaminating fibroblasts while limiting the use of cytotoxic reagents such as cytosine arabinoside (ara-C) [34]. These methods take advantage of fluorescence-activated cell sorting (FACS) [35] and the differing adherent properties of human SCs and fibroblasts [35,36,37,38]. Despite this, the attempts to increase purity in cell cultures often result in low yields of SCs [35,39]. The initial dissociation, seeding, and sorting of human SCs can also take a substantial amount of time [37,38], which would be problematic for large-scale expansion and automation. Expanding these primary cell cultures for use in clinical trials represents another issue as often at least eight weeks of culturing can be required to obtain sufficient numbers just for in vitro research [37]. This is achieved using flasks coated with expensive reagents including laminin, poly-L-ornithine, and poly-L-lysine and using complex media containing additional growth factors with short half-lives [35,36,38,39,40]. Additionally, primary human SCs only proliferate for a limited number of passages [41] before becoming senescent.

Alternative sources of SCs from reliable, GMP-grade origins that can expand to sufficient cell numbers in a scalable way will be required if they are to be developed further for clinical applications.

## 4. Alternative Sources of Schwann Cells

As mentioned above, SCs are such an integral part of peripheral nerve repair, but primary human SCs are a challenging source of cells for nerve tissue engineering [32,33]. Conclusively, there has been a drive for the development of robust protocols for the differentiation of SCs from a wide range of stem cells.

Both embryonic and adult stem cell sources have been reviewed elsewhere [42,43,44,45,46] and various SCs differentiation protocols have been developed with some success. For example, SCs differentiated from embryonic stem cells (ESCs) express SC markers and associate with axons in vitro, suggesting myelination ability [47]. Although ESCs proliferate rapidly, there are ethical [48] and safety [49] concerns in using ESCs which outweigh the advantages when progressing to cell therapy. Pluripotent human ESCs can be derived from the inner cell mass of a blastocyst (embryo prior to placental implantation, at about four days after fertilization) that has not yet differentiated into one of the three germ layers. Although embryos can be derived from in vitro fertilization and methods have been proposed for deriving hESCs from embryos in the four-cell stage, increasing the probability of embryo survival, ethical concerns remain for the use of hESCs [48]. With regard to safety concerns, the risks of teratoma formation and local overgrowth after transplantation as well as the risk of becoming cancerous or infected are still relevant and the topic of current research [50]. Adult stem cell sources such as bone marrow, adipose, and umbilical cord mesenchymal stem cells [51,52,53,54,55,56,57], and those from more superficial locations such as skin-derived precursor stem cells (SKP-SCs) [58,59], epidermal neural crest stem cells [60], and dental pulp stem cells (DPSCs) [61,62] have also been shown to successfully differentiate into SCs. However, these and other adult stem cell sources proliferate slowly, require invasive harvesting procedures, are variable in quality, and may have potential to differentiate into a range of cell types other than neurons and glia.

Despite this broad selection of sources and literature available on a diverse range of options that can be used to derive SCs, no stem cell-derived SCs have yet progressed to clinical trials, largely because of complications around availability, control, safety, and ethical restrictions. Induced pluripotent stem cells (iPSCs) overcome many of the limitations presented when ESCs and adult stem cells are used as sources of SCs. The origin of iPSCs is ethically less challenging, since they are mainly derived from genetically reprogrammed skin fibroblasts, peripheral blood mononuclear cells (PBMCs), or even cord blood cells [63]. Of the different sources, PBMCs represent the most advantageous cell type since they can be collected during minimally invasive blood extraction and can afterwards immediately be reprogrammed [63]. Safe reprogramming is achieved by transduction with viruses that do not permanently integrate into the host cell genome [63]. The resulting iPSCs can self-renew, proliferate rapidly, and can differentiate into any cell type of the three germ layers (endoderm, mesoderm, and ectoderm) [50,64]. Additionally, they can be used without the ethical restrictions that surround the use of ESCs [50,64] and can be GMP-grade [65,66].

Although no peripheral nerve injury clinical trials have started using iPSC-derived SCs, clinical trials using iPSCs are mainly driven by research in Japan [67,68], emerging at an increasingly rapid pace. Recently, a systematic multi-database analysis was published reviewing worldwide trends for using pluripotent stem cells [69]. When focussing on neurodegenerative or neurotraumatic conditions, the application of iPSC-derived nervous system cells in clinical trials mainly includes retinal pigment epithelium cells for treating age-related macular degeneration [70,71] or dopaminergic progenitor cells for treating Parkinson’s disease [72,73,74]. Another frequently investigated approach is cell therapy for treating spinal cord injury, and actually here also primary human SCs are considered as a valuable tool [75]. With regard to iPSC-derived cell replacement in clinical spinal cord injury repair, not SCs but neural stem or progenitor cells have been considered so far [75,76]. For the use of any iPSC-derived cell replacement, allogeneic cell therapies are a more attractive option than autologous therapies when cost is considered, with banks of iPSC lines matched to the range of human leukocyte antigen (HLA) variability within a population as a possibility to address immunogenicity [66,77]. In this regard, the feasibility of creating a genomic stability-validated iPSC bank containing homozygous cell lines to allow HLA matching for a large number of potential recipients is being widely explored [78], and would also facilitate allogenic cell replacement in nerve tissue engineering.

To mitigate the potential risk of genomic instability and accumulation of oncogenic mutations [65,79], any iPSC therapy must, however, be extensively genetically screened prior to use [48,50] and any undifferentiated cells need to be detected by robust methods [80]. 

## 5. Induced Pluripotent Stem Cells as a Source for Schwann Cells

During embryonic development, SCs originate from the neural crest cells which migrate to the periphery and differentiate into Schwann cell precursors (SCPs) before maturing into non-myelinating and myelinating SCs [3,81]. Therefore, all methods of differentiating iPSCs to SCs pass through an intermediate progenitor cell stage—either neural crest stem cells or SCPs [81]. There is an argument that SCPs are neural crest stem cells, or “proto-neural crest stem cells”, due to their morphological and phenotypic similarities [82], but for the purpose of this review, we will consider neural crest stem cells and SCPs as independent cell types as defined in the protocols to be discussed below. Neural crest cells or neural crest stem cells are a migratory, transient population of cells originating at the neural plate [83], from which SCPs develop before transitioning to immature and then mature SCs [84]. Kim et al. distinguished between these two cell populations by gene expression analysis, for example, PAX3 and TWIST are marker genes for neural crest stem cells, NGFR and SOX10 for neural crest stem cells/SCPs, and GAP43 and MPZ are markers for SCPs [85].

Early iterations of differentiation protocols which were targeted to peripheral nerve injury repair involved differentiating iPSCs to neural crest stem cells and implanting the cells at injury sites at this stage. Schwann cells were found to have differentiated from the implanted cells in vivo [86,87]. It is noteworthy, however, that in Okawa et al. [86], transplantation was executed by injection of the cells into hind limb skeletal muscles and that vascular smooth muscle cells could also differentiate from the stem cell source. The differentiation potential of neural crest stem cells was further demonstrated in vitro by Kreitzer et al., with neural crest stem cells spontaneously differentiating again into smooth muscle cells, peripheral neurons, and SCs as characterized by immunocytochemical detection of SMAα, peripherin, and GFAP expression, respectively [88].

Although these studies are evidence that iPSCs can differentiate into SCs via an intermediate neural crest stem cell stage, these rather undirected or spontaneous methods of SC differentiation are likely to be unsuitable for use in clinical cell therapy due to the unpredictability of the differentiation and the low yield and purity of SCs in the final cell population.

## 6. Methods to Differentiate iPSCs to Schwann Cells

For a closer look on more directed iPSCs differentiation protocols, only those studies using iPSCs as the initial cell type before differentiation and which subsequently identified SCs in vitro or in vivo have been included in the current review. All, except one which uses mouse cells [86], start with human iPSCs.

**Figure 1:**
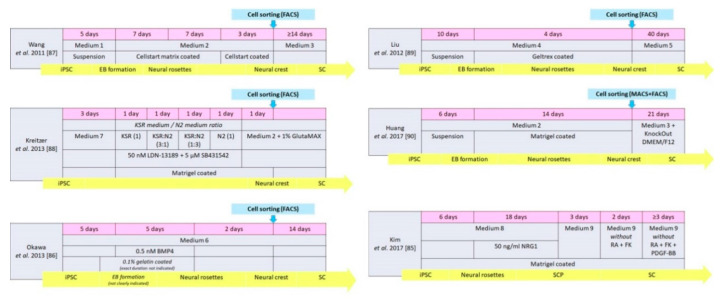
Timing, conditions, and stages for the differentiation of induced pluripotent stem cells (iPSC) to Schwann cells (SC) according to different protocols. Pink bar indicates the length of time for each stage (the media used are summarized in line 2, with more information on the components provided in Table 1). Whether the cells are cultured in suspension or on a coated surface, as well as the type of coating used, can be seen in the bottom gray line. The yellow bar indicates the cells identified to be present at each stage as defined in the respective protocols. If any cell sorting is included, this is shown by the blue box and arrow. Abbreviations: BMP4: bone morphogenetic protein 4. DMEM/F12: Dulbecco’s modified Eagle’s medium/Nutrient Mixture F-12 Ham. EB: embryoid bodies. FK: forskolin. KSR and N2 medium according to Kreitzer et al. [88], see Table 1. NRG1: neuregulin 1. PDGF-BB: platelet-derived growth factor two B subunits. RA: retinoic acid. SCP: Schwann cell precursors.

As illustrated in Figure 1, there are two main starting culture types for the stepwise differentiation of iPSCs into SCs—either the cells start as embryoid bodies (EB) [89,90] or already as a monolayer culture [85], likely derived from a not clearly indicated EB stage [91]. Both sources give origin to pluripotent stem cells and need to go through intermediate stages, e.g., neural rosettes, neural crest cells, or SCPs, before culture conditions can further be adjusted to differentiate these cells into SCs. Intermediate stages, however, are not characterized in detail in most of the SC-specific protocols. The total length of time required to progress from the iPSC stage to the intermediate stage and on to the SCs stage is compared for six different published protocols in Figure 1. The figure additionally details the cell type, culture media, and culture surface coating used. Greater detail on media components is listed in Table 1, while Table 2 summarizes the differentiation protocols and the cells identified as well as the markers and methods used in identifying the differentiated cells. Any Schwann cell functionality tests, if undertaken, can be seen in the final column of Table 2.

The total length of time to differentiate iPSCs to SCs varies from 31 to 54 days. Two methods differentiate the cells to the neural crest stage before the cells are differentiated further spontaneously in vitro [86], or after transplantation [88]. The proteins chosen to identify the cell types present at each stage are similar between the methods, with Sox10 and AP2α representing the most commonly used markers for the intermediate cells (neural crest stem cells or SCPs) and S100β, GFAP, and p^75NGFR^ representing glial cell markers for differentiated SCs. There is limited data available on differentiated SC functionality, with this either not being tested in the studies [86,87,88] or using in vitro myelinating cultures with rat DRG in one study [89]. Only two studies [85,90] additionally tested differentiated SCs’ functionality in vivo in peripheral nerve injury models.

The protocols published by Liu et al. [89] and Huang et al. [90] are most similar to each other, starting with embryoid bodies and progressing through each cell stage at a similar rate. The neural crest induction medium [89] and neural crest stem cell medium [90] used have some similarities with both containing fibroblast growth factor-2 (FGF2) and being serum-free. However, other aspects such as the base medium and length of time of induction (10 days in Liu et al. [89] and 6 days in Huang et al. [90]) are dissimilar and are summarized in Table 1; Table 2, respectively. Detail on media components can be found in Table 1, and more information on the time spent at each differentiation stage in Figure 1.

The protocol in Kim et al. [85] differs to the other protocols in that iPSC colonies are firstly plated directly on Matrigel with no preceding embryoid body stage. The neural crest differentiation media in Kim et al. [85] are also very different to the other two protocols mentioned before [89,90] as they contain two signaling pathway inhibitors, CT99021 and SB431542. SB431542 is an inhibitor of the activin-receptor-like kinase (ALK) receptors ALK5 and 7 and acts via the TGF-β/Activin/NODAL pathway, and CT99021 is a Wnt signaling pathway activator which inhibits the kinase GSK3. Switching off the pluripotency genes in the iPSCs and adding neuregulin protein earlier on in the process may be why the authors reported such high yields of Sox10-positive cells (a transcription factor expressed by those in the Schwann cell lineage) in their cultures and why they did not need to sort their cultures for differentiated SCs [85]. This contrasts with the two previously discussed methods [89,90]. The length of time needed for differentiating cells from the iPSC stage to the SCs stage is much shorter in the Kim et al. protocol, taking around 32 days in total [85], compared to the protocols published by Liu et al. (~54 days, [89]) and Huang et al. (~41 days, [90]).

In general, shorter protocols, as reported by Kim et al. [85], would be beneficial when looking ahead at the expansion of cells for future regenerative medicine cell therapy approaches. Their expandable SCPs with potential to differentiate to both SCs and melanocytes were also shown to be stable in terms of gene expression at up to 100 passages [85], again very useful for potential cell therapy use which requires expandable cells of a consistent quality.

A limitation to all of the reported methods is that no SCs were cultured for longer than two–three weeks since being used in animal peripheral nerve injury models at this point. According to our best knowledge, it has not been demonstrated so far whether the differentiated cells at the Schwann cell stage are able to be further expanded in the same way as neural crest stem cells or Schwann cell precursors. This would, however, be beneficial for their use in regenerative medicine approaches.

## 7. Characterizing Schwann Cells Differentiated from iPSCs

In order to use cells in therapeutic applications, they must be characterized extensively to both identify the cells used but also to ensure that the respective cell populations do not include potentially harmful cell types. Whole-genome analysis will need to be undertaken to detect any potential infiltrating mutations which could alter the phenotype (for example, oncogenic mutations) when any cell differentiated from stem cells is used as a therapy. It is also critically important to understand the genotype and phenotype of the differentiated cells.

Huang et al. [90] only described immunocytochemical detection of two glial markers in their differentiated SCs—glial fibrillary acidic protein (GFAP) and S100β. Activated astrocytes of the central nervous system are also GFAP-positive [92,93] and therefore are not a definitive marker for SC differentiation.

Both Liu et al. [84] and Kim et al. [85] used whole-genome microarrays and compared the genome similarity of primary human SCs to their differentiated SCs. Liu et al. [89] compared the genome to that of immortalized foetal SCs, rather than adult ones, while the primary human SCs used by Kim et al. [85] were isolated from human spinal nerves. Both groups found the expression profiles of the differentiated SCs closely resembled those of either the immortalized human foetal [89] or human spinal nerve [85] SCs selected for comparison. As adult human peripheral nerves were not used as a control, it was not proven how similar to peripheral nerve SCs the differentiated cells were.

All three groups compared the differentiated SC gene expression, protein expression, and neurotrophic factor expression and release to that of the progenitor-stage cells [85,89,90]. All differentiated SCs expressed neural crest stem cell markers such as p75^NGFR^ but had higher expression levels of SC-specific markers such as GFAP, S100β, early growth response 2 (EGR2), and proteolipid protein (PLP).

In addition, Huang et al. [90] and Kim et al. [85] looked at the expression of neurotrophic factors. This is, as mentioned before, an important feature as SCs not only support axon regeneration through direct contact, but also indirectly through the release of neurotrophic factors. As detected by ELISA, transplanted neural crest stem cells in Huang et al. [90] increased the amount of NGF and BDNF in the surrounding tissues. In comparison, the SCs generated by Kim et al. [85] demonstrated an increased expression level of NGF, GDNF, and BDNF in vitro compared to SCPs, which was also confirmed by ELISA performed on respective conditioned media. However, Kim et al. [85] did not examine neurotrophic factor release in the tissue surrounding the rat sciatic nerve injury in their in vivo evaluation. Therefore, it is unknown if the increased expression in vitro also corresponded to a greater concentration of the neurotrophic factors in the nerve bridge.

Overall, we must conclude from the studies discussed above that more thorough analysis of differentiated SCs and comprehensive characterization of their properties in vivo [94] will be needed prior to any translation into clinical use in cell therapies.

## 8. Phenotype of Differentiated Schwann Cells—In Vitro and In Vivo

A further drawback with regard to a future translation into clinical use of the studies that have so far analyzed the potential of iPSC-derived SCs in rodent peripheral nerve repair models is that an autograft (the “gold standard” treatment for long-gap peripheral nerve injuries [5]) was not used as a standardized control.

Exclusively Huang et al. [90] and Kim et al. [85] evaluated their iPSC-derived cells in vivo, however, as also different peripheral nerve injury models were used, the functional results cannot be directly compared. Adult female athymic nude rats with a 1 cm sciatic nerve gap were used as a model in the Huang et al. study [90], and 8-week-old C57BL/6 male rats with a short 2–3 mm nerve defect were analyzed by Kim et al. [85]. In addition to the use of different models, the method of implanting the differentiated cells varied between the two studies. Cells were suspended in a collagen/hyaluronic acid hydrogel filled into a poly(L-lactide-co-caprolactone) (PLCL) conduit by Huang et al. [90]. On the contrary, Kim et al. [85] injected their cells suspended in Matrigel directly at the injury site. The chosen control conditions also differed, with Huang et al. [90] comparing acellular conduits with conduits containing either neural crest stem cells or differentiated SCs, and Kim et al. [85] comparing an injection of Matrigel with SCs with acellular Matrigel solution.

The choice of the animal model may account for the reported differences in the success of the differentiated SCs supporting axon regrowth. Kim et al. [85] found the differentiated SCs supported regeneration and remyelination to a greater degree compared to Matrigel alone, whereas Huang et al. [90] concluded that their neural crest stem cells performed better than their differentiated SCs when looking at functional parameters such as compound muscle action potential (CMAP) recovery rate, although other parameters compared between conduits containing neural crest stem cells and SCs were similar. The neural crest stem cells also migrated further along the conduit than the SCs, which were found to be clustered at the proximal end [90]. This is likely to be linked to the migratory behavior of neural crest stem cells seen during development [95] and the larger nerve gap in the animal model used by Huang et al. [90]. The majority of neural crest stem cells were additionally found to have differentiated further once implanted, into both SCs (>70%) and fibroblasts (around 20%) after one month in vivo [90]. Despite the regenerative potential, it could be a risk to use these cells for therapeutic applications as with the ability to differentiate further comes the potential of differentiation down an inappropriate lineage. Huang et al. [90] also found that their neural crest stem cells performed better than their differentiated SCs in terms of supporting regeneration and increasing axon counts, although this may be due to better engraftment compared with differentiated SCs.

High yields of the desired cell type are required when developing cell therapies for future clinical use. Although sorting differentiating cells tends to increase the proportion of the desired cells in the total population compared to undifferentiated cells, it can reduce the overall yield. Liu et al. found 78% and 85% of the differentiated SCs expressed GFAP or S100β, respectively [89]. While Kim et al. did not sort the cells at any point, they found 99% of the Schwann cell precursors were positive for the SC lineage marker Sox10 [85]. However, while the expression level of the mature SC marker S100β was higher in the differentiated SCs compared to the Schwann cell precursors, the proportion is not known [85]. Again, as already discussed above, it is noteworthy considering that it is unclear how similar the differentiated SCs investigated were to human peripheral nerve SCs.

## 9. Conclusions

Schwann cells play a critical role in peripheral nerve repair through axon guidance and promoting the establishment of a pro-regenerative environment in the nerve bridge. However, primary SCs may not be ideal for efficient use in cell therapies, due mainly to difficulties in purifying and a long expansion process. Despite extensive efforts to develop reliable methods to differentiate stem cells to SCs as an alternative source, both adult stem cells and ESCs have drawbacks—from low purity and yield, non-neuronal differentiation potential, and accessibility of cells, to ethical considerations. Although therapies using differentiated cells sourced from iPSCs will require extensive screening prior to use, iPSCs have the benefits of ESCs without the drawback of ethical concerns which have been cited as a potential barrier to the application of ESC therapies worldwide.

There are several protocols differentiating iPSCs to neural crest stem cells, with the three studies looked at in the current review [85,89,90] including a step to differentiate progenitor cells to SCs, showing promising outcomes both in vitro and in vivo. All three methods generate differentiated cells that express SC markers and release neurotrophic factors. A clear comparison of in vivo outcomes on peripheral nerve repair cannot be made due to the varied choice of animal models used.

It would be beneficial to compare the cell types derived from the different protocols in similar animal models of peripheral nerve injury, the true determinant being how well they perform compared to the autograft in a critical length gap and comprehensive functional analysis. Despite this, the use of iPSCs as a source for Schwann cells for use in future peripheral nerve injury repair therapies remains very promising.

## Figures and Tables

**Table 1:** Components of the various media used in the selected differentiation protocols [85,86,87,88,89,90]. Many have similar base media, e.g., knockout (KO) DMEM/F12, as well as components routinely found in neuronal differentiation cultures such as N2 and B27. GlutaMax is a more stable form of L-glutamine. The addition and concentration of additional growth factors varies substantially between protocols.

Medium 1	Medium 2	Medium 3	Medium 4	Medium 5	Medium 6
80% KO DMEM/F12, 20% KSR, 1% NEAA 1 mM L-Glu 0.1 mM 2-ME	KO DMEM/F12, 2% StemPro neural suppl., 20 ng/mL FGF-2, 20 ng/mL EGF	N2 M., 10 ng/mL CNTF, 10 ng/mL FGF-2, 1 mM dBcAMP, 20 ng/mL NRG1	50% (Neurobasal M., MEM-NEAA, GlutaMAX, B27, 20 ng/mL FGF-2) + 50% (SDIA condition M, 10 μM Y-27632, 200 μM AA)	MesenPRO M., 20 ng/mL NRG1	SDIA condition M., 10% KSR, 0.1 mM NEAA, 1 mM pyruvate, 0.1 mM 2-ME
**Medium 7**	**Medium 8**	**Medium 9**	**according to Kreitzer et al.** [88]		
			**KSR medium**		**N2 medium**
DMEM/F12, 20 ng/mL FGF-2, 1% N2, 2% B27, 0.05% BSA fraction V, 1% GlutMax, 1% MEM-NEAA, 110 µM 2-ME, 10 µM Y-27632	Advanced DMEM/F12 + Neurobasal M. (1:1 mix), 1% N2, 2% B27, 0.005% BSA, 2 mM GlutaMax, 0.11 mM 2-ME, 3 mM CT99021, 20 mM SB-431542	DMEM/low glucose, 1% FBS, 4 mM FK, 200 ng/mL NRG1, 100 nM all-trans RA, 10 ng/mL PDGF-BB	Knockout DMEM, 15%KSR, 1% MEM-NEAA, 1% GlutaMax, 55 µM 2-ME		DMEM/F12, 0.15% glucose, 1% N2, 20 µg/mL insulin, 5 mM HEPES

AA: ascorbic acid. B27: B-27™ Supplement. BSA: bovine serum albumin. CNTF: ciliary neurotrophic factor. CT99021: 6-((2-((4-(2,4-Dichlorophenyl)-5-(4-methyl-1H-imidazol-2-yl)pyrimidin-2-yl)amino)ethyl)amino)nicotinonitrile. dBcAMP: dibutyryl cyclic adenosine monophosphate. EGF: epidermal growth factor. FBS: fetal bovine serum. FGF-2: basic fibroblast growth factor. FK: forskolin. HEPES: 4-(2-hydroxyethyl)-1-piperazineethanesulfonic acid. KSR: knockout serum replacement. L-Glu: L-glutamine. M: medium. N2: N-2 Supplement. NEAA: non-essential amino acid. NRG1: neuregulin 1. PDGF-BB: platelet-derived growth factor two B subunits. SB-431542: 4-[4-(1,3-benzodioxol-5-yl)-5-(2-pyridinyl)-1H-imidazol-2-yl]benzamide. SDIA: stromal cell-derived inducing activity. Suppl: supplement. 2-ME: 2-mercaptoethanol. Y-27632: (1R,4r)-4-((R)-1-aminoethyl)-N-(pyridin-4-yl)cyclohexanecarboxamide.

**Table 2:** Summary of induced pluripotent stem cells (iPSC) to Schwann cells (SC) differentiation protocols. The table can be split between the progenitor cell stages (columns 3–6) and the Schwann cell stage (columns 7–11). The key markers used to identify the differentiated cell types include common Schwann cell markers such as Sox10, GFAP, S100β, and p^75NGFR^ as well as neural crest cell markers AP2 and Slug. Information on whether the differentiation from the progenitor to the SC stage was direct or spontaneous is included in column 8. If the Schwann cells’ functionality was tested can be seen in the very right column (In vitro: myelinating culture with rat DRG neurons. *In vivo*: seeded in nerve guidance conduit in a rat sciatic nerve injury model). Only two studies [85,90] included in vivo testing of their iPSC-derived SCs.

			Progenitor Cell Stages			Schwann Cell Stages				
Study	Source iPSC	Culture Condition	Type	Duration (days)	Cell Markers	Induction Medium	Type of Induction	Duration (days)	Cell Markers	Functionality Analysis
**Wang** **et al. 2011 [87]**	Human	Medium 1 + 2	Neural crest	22	p75^NGFR^, HNK1 Vimentin, Nestin Slug, AP2α	Medium 3	Directly	≥14	S100β, GFAP	None
**Liu** **et al. 2012 [89]**	Human	Medium 4	Neural crest	14	p75^NGFR^, HNK1, Sox9, Sox10, CD44	Medium 5	Directly	40	GFAP, S100, p75^NGFR^	In vitro
**Kreitzer** **et al. 2013 [88]**	Human	Medium 7, KSR medium, N2 medium, Medium 2, GlutaMAX	Neural crest	8	p75^NGFR^, HNK1 AP2α, Sox10	Medium 2 GlutaMAX	Spontaneous in vitro	*Not mentioned*	GFAP	None
**Huang** **et al. 2017 [90]**	Human	Medium 2	Neural crest	20	HNK1, AP2α, Sox10	Medium 3 KnockOut DMEM/F12	Directly	21	S100β, GFAP	*In vivo*
**Kim** **et al. 2017 [85]**	Human	Medium 8	SC precursor	24	Sox 10, CDH19, MPZ, GAP43	Medium 9	Directly	≥ 7	NGFR, S100, EGR2, MPZ	In vitro and *in vivo*
**Okawa** **et al. 2013 [86]**	Mouse	Medium 6 BMP4	Neural crest	12	p75^NGFR^, AP2α	Medium 6	Spontaneous *after implantation*	14	S100β	None

AP2α: activating enhancer binding Protein 2 alpha. BMP4: bone morphogenetic protein 4. CDH19: Cadherin 19. EGR2: early growth response protein 2. GFAP: glial fibrillary acidic protein. HNK1: human natural killer-1. MPZ: myelin protein zero. NGFR: nerve growth factor receptor. S100/S100β: calcium binding protein.
